# Ulcerative Colitis: Novel Epithelial Insights Provided by Single Cell RNA Sequencing

**DOI:** 10.3389/fmed.2022.868508

**Published:** 2022-04-20

**Authors:** Joao M. Serigado, Jennifer Foulke-Abel, William C. Hines, Joshua A Hanson, Julie In, Olga Kovbasnjuk

**Affiliations:** ^1^Division of Gastroenterology and Hepatology, Department of Internal Medicine, University of New Mexico Health Sciences Center, Albuquerque, NM, United States; ^2^Division of Gastroenterology and Hepatology, Department of Medicine, Johns Hopkins University School of Medicine, Baltimore, MD, United States; ^3^Department of Biochemistry and Molecular Biology, University of New Mexico Health Sciences Center, Albuquerque, NM, United States; ^4^Department of Pathology, University of New Mexico Health Sciences Center, Albuquerque, NM, United States; ^5^Department of Microbiology and Immunology, University of Maryland School of Medicine, Baltimore, MD, United States

**Keywords:** Ulcerative Colitis, single cell RNA sequencing, intestinal epithelium, goblet cells, colonic microfold cells, stem cells

## Abstract

Ulcerative Colitis (UC) is a chronic inflammatory disease of the intestinal tract for which a definitive etiology is yet unknown. Both genetic and environmental factors have been implicated in the development of UC. Recently, single cell RNA sequencing (scRNA-seq) technology revealed cell subpopulations contributing to the pathogenesis of UC and brought new insight into the pathways that connect genome to pathology. This review describes key scRNA-seq findings in two major studies by Broad Institute and University of Oxford, investigating the transcriptomic landscape of epithelial cells in UC. We focus on five major findings: (1) the identification of BEST4 + cells, (2) colonic microfold (M) cells, (3) detailed comparison of the transcriptomes of goblet cells, and (4) colonocytes and (5) stem cells in health and disease. In analyzing the two studies, we identify the commonalities and differences in methodologies, results, and conclusions, offering possible explanations, and validated several cell cluster markers. In systematizing the results, we hope to offer a framework that the broad scientific GI community and GI clinicians can use to replicate or corroborate the extensive new findings that RNA-seq offers.

## Introduction

Ulcerative Colitis (UC) is a chronic and debilitating inflammatory disease of the colon, and is a distinct condition within a broad group of pathologies termed inflammatory bowel disease (IBD), which includes Crohn’s disease (CD) and indeterminate colitis. The etiology of UC remains uncertain, but increasing evidence implicates complex genetic and environmental contributions. Early genomic and immunologic studies were crucial to identifying therapeutic targets and avoiding surgical intervention. Development of infliximab and other monoclonal antibodies against TNFα, and the biologics targeting cytokines (IL-12 or IL-23) that significantly reduce inflammation and induce macroscopic/endoscopic healing, were major breakthroughs in the management of UC (1). Newer drugs such as anti-integrin agents (vedolizumab and ustekinumab) and JAK inhibitors also yielded endoscopic improvements (1–4).

With the development of biologic therapies, the goal of UC treatment extended beyond symptomatic improvement to include both histologic and molecular healing. Several observational studies have shown that histological remission, including epithelial tissue restoration, is associated with lower rates of disease-related complications such as hospitalization, corticosteroid use, and colectomy compared to either resolution of symptoms or endoscopic improvement alone (5–7). Recognizing that histologic improvements are now included in UC clinical trials of multiple novel therapeutic agents (8), markers of active disease, therapeutic response and remission are urgently needed.

There are numerous UC-related pathologies that contribute to inadequate epithelial maintenance and regeneration. Cell turnover is increased due to autophagy and apoptosis, yet colonocyte differentiation is reduced (9–11). Barrier defects manifest as abnormal glycosylation and sulfation of mucins, loss of the protective mucus layer, and increased tight junction permeability (12–16). Microbial dysbiosis also contributes to altered epithelial function, in which reduced butyrate oxidation yields energy-deficient epithelium, and increased exposure to microbial signals stimulates inflammatory cytokine secretion (17–20). It is not clear if the many recognized pathologies initiate the disease or are consequences that fuel further development. Overall, the spectrum of pathologies highlights the challenges in defining molecular markers of health, UC damage, and epithelial restoration.

Single cell RNA sequencing (scRNA-seq) allows for in-depth transcriptional characterization of epithelial pathologies in UC and identification of possible pharmacologically fruitful targets. In this review, we discuss findings from two independent scRNA-seq datasets generated by the Broad Institute (21) and the University of Oxford (22) that compared subsets of epithelial cell types in healthy and UC colonic tissue. Our goal is to highlight novel markers for epithelial UC disease that, when further validated, may lead to identification of pharmacologic targets to promote epithelial healing in UC patients.

## Epithelial Cell Diversity Requires Single Cell Resolution to Capture Disease Markers

The heterogeneity of cells that compose the normal colonic epithelium originates from a common intestinal stem cell through the actions of multiple niche factors. As cells migrate from the crypt to surface epithelium, they mature into specialized cells for molecular transport, sensing, secretion, and barrier formation. Each epithelial cell type is critical to sustain organ function, and perturbations in the proportion or phenotype of a cell lineage can contribute to disease. Thus, approaches to study epithelial cell identity and function must distinguish among unique populations within the epithelium.

Transcript analyses using mRNA microarrays that preceded RNA-seq analysis allowed for the simultaneous detection of thousands of genes at one time (23) and was essential to demonstrate the presence of a persistent inflammatory state in patients with histologic remission (24, 25). A significant disadvantage of the microarray is that they require prior knowledge of the gene sequence under investigation. In contrast, RNA-seq has a wider dynamic range, allows for sequencing of transcripts without prior information, and can detect gene fusions, indels, and single nucleotide polymorphisms (26). scRNA-seq captures detailed molecular snapshots of individual cells that can be compared between healthy and diseased states to identify altered cell populations within non-homogeneous tissues. scRNA-seq quantifies the expression of each individual gene at a single cell level, offering high sensitivity for low abundance targets that may be masked in a bulk sequencing approach. This technology unveils the heterogeneity within major cell types, describes cell clusters that contribute to the pathogenesis of disease, and allows for a detailed description of the pathways that connect genome and transcriptome to pathology (27). scRNA-seq might be ideal to understand epithelial pathologies associated with UC in relation to healthy cells.

The technical aspects of scRNA-seq are described in great detail in several excellent reviews (28–30). Typical elements of scRNA-seq analysis include single-cell dissociation, isolation, library construction, and single cell sequencing (27, 31, 32). In general, the datasets generated by this technique are large and highly complex requiring robust bioinformatic analysis to be fully interpretable (29, 33).

The complexity of genetics and environmental factors that fuel UC make it a particularly good candidate for investigation through single cell transcriptomics. scRNA-seq potentially allows for detailed molecular snapshots of the different cell types composing the intestinal mucosa in health and UC. In particular, this technology facilitated the identification of previously unrecognized cell types and their potential transcriptional shift in disease that correlated to disease severity. These new findings could have direct implications in the management of UC. By stratifying patients on the basis of underlying transcriptional and genetic variability, scRNA-seq opens new ways for the development of targeted therapies.

A comparison of parameters and outcomes in the Broad and Oxford scRNA-seq studies is highlighted in Table 1. Notably, the number of subjects sampled and the epithelial cell types, or clusters identified by the two studies, differs. We focus on cell clusters that exhibited most dramatic transcriptional changes with potential relevance to inflammation, host stress response, immune regulation, and epithelial regeneration: BEST4^+^ cells, colonic M-like cells, goblet cells, colonocytes and stem cells.

**TABLE 1:** Summary of RNA-seq studies in Ulcerative Colitis.

	Broad study	Oxford study
Study subjects	18 patients with UC and 12 healthy controls	3 patients with UC and 3 healthy individuals
Biopsy samples	One non-inflamed and one inflamed region or Two adjacent non-inflamed and two adjacent inflamed biopsies	Endoscopically inflamed distal areas of the colon and proximal clinically non-involved regions
Number of cells yielded	366,650 cells	11,175 cells
Single cell suspension	Dissociation with TrypLE Express Enzyme	Dissociation with TrypLE Express Enzyme
RNA-seq methodology	Droplet-based scRNA-seq technique	Droplet-based scRNA-seq technique
Library preparation	SMART-Seq2 protocol	SMART-Seq2 protocol
Differential gene expression	MAST; AUC ≥ 0.75	Seurat; AUC ≥ 0.7
Cells identified	Identified fifteen distinct groups of epithelial cells: - Stem cells - TA 1 - TA 2 - Cycling TA - Immature Enterocytes 1 - Immature Enterocytes 2 - Enterocyte Progenitors - Enterocytes - Microfold cells - BEST4 + Enterocytes - Secretory TA - Immature Goblet cells - Goblet cells - Tuft cells - Enteroendocrine cells	Identified seven distinct groups of epithelial cells*: - Undifferentiated #1 - Undifferentiated #2 - Enteroendocrine cells – Colonocytes - Crypt Top Colonocytes - BEST4 + /OTOP2 cells - Goblet cells

*TA: transit amplifying.*

*Which were further subdivided into 5 clusters based on their location along crypt-surface axes.

## Best4^+^ Cells

**FIGURE 1:**
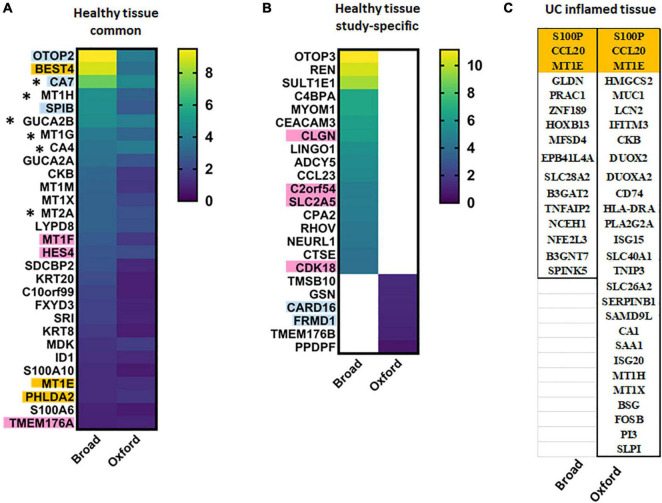
BEST4^+^ cell characteristic transcripts in health and UC inflammation. Heat maps of transcripts significantly elevated in BEST4^+^ cells in **(A)** both studies or **(B)** one of the studies in healthy epithelium presented as log_2_[fold change (FC)]; **(C)** transcripts characteristic for BEST4^+^ cells in UC inflamed epithelia. Transcripts in orange are BEST4^+^ cell-specific in both studies and thus might serve as cluster markers in health or disease. Transcripts in blue are specific to healthy BEST4^+^ cells in Oxford study; transcripts in pink are specific to healthy BEST4^+^ cells in Broad study. * – transcripts are elevated in healthy BEST4 cells in both studies, but are downregulated in active UC in the Broad study.

Both data sets discovered a novel differentiated absorptive surface colonocyte cluster characterized by expression of a Ca2**^+^**-sensitive chloride channel (BEST4) and proton-conducting ion channel (OTOP2) proposed to regulate luminal pH. These cells were also enriched in the paracrine hormones guanylin (GUCA2A) and uroguanylin (GUCA2B), which can stimulate fluid and electrolyte transport through action on the receptor guanylate cyclase. Among 46 transcripts significantly elevated in healthy BEST4 + cells in the Broad study, 29 were also detected as significantly elevated in the Broad study (Figure 1). However, several transcripts were not exclusive to BEST4 + cells and thus do not serve as unique markers.

We performed STRING interactome analysis (human Protein-Protein Interaction Networks and Functional Enrichment Analysis using^1^) of the characteristic transcripts of BEST4 cells which confirmed across both studies that in healthy tissue, BEST4 cell function includes metallothionein expression (HSA-5661231), guanylate cyclase/dehydratase activity (GO:0030250), and proton channel activity (GO:0015252). Surprisingly, proteomic analysis of these BEST4 cells, conducted in the Oxford study, identified several highly upregulated proteins as BAG1 and APOO. These transcripts were not identified as “characteristic” for this cell cluster in Oxford scRNA-seq dataset (the exceptions were BEST4 and CTSE proteins which were only slightly upregulated in comparison to the rest of epithelial cells). These data indicate that further validation at the protein levels are necessary to reliably identify these BEST4 + cells in tissue and better define their role in colonic physiology.

UC-associated changes in the BEST4 + cell transcriptome did not completely agree between the studies. The Broad study found the number of BEST4 + cells were substantially reduced in UC, and 16 significantly upregulated transcripts (log_2_FC ≥ 4.0; Figure 1) were putative disease-associated BEST4 cell markers. In contrast, the Oxford study observed stable numbers of BEST4 + cells and listed 28 upregulated transcripts (fold increase for each transcript was not provided) of which only two (CCL20 and S100P) overlapped with the Broad results (Figure 1). MT1E was the only transcript elevated in BEST4 + cells in both studies regardless of disease state. Other UC-associated BEST4 + upregulated transcripts, such as MT1H and MUC1 (Oxford) or TNFAIP2 and NCEH1 (Broad), did not correlate between the studies. Results of the Oxford study suggest that BEST4 + cells maintain host protective functions in UC [metallothioneins (HSA-5661231), antibacterial (HSA-6803157) and antiviral (CL:4665) pathways], whereas no specific function was identified in the elevated transcripts from Broad study.

Because most of the upregulated transcripts in UC-associated BEST4 + cells were elevated in other cells types, we cross-referenced the single cell results with an earlier bulk RNA-seq data set generated from three treatment-naïve UC patients (34). Markers of healthy BEST4 + cells, including BEST4, OTOP2, CA7, MT1H, and MT1G, were also downregulated in active UC analyzed by bulk RNA-seq. Elevation of UC-associated transcripts not specific to BEST4 + cells, including CCL20, S100P and PLA2G2A, were also detected in the bulk RNA-seq study. Upregulated PLA2G2A expression was further confirmed by qRT- PCR and immuno-histochemistry in tissue samples from UC patients and healthy controls, further supporting the bulk RNA-seq and scRNA-seq data (34).

Additional scRNA-seq study of colon biopsies from UC patients and healthy controls of Chinese Han ancestry (35) complemented the Broad and Oxford studies that were conducted in individuals of primarily European ancestry (21, 22). In this study, 43,218 cells representing UC-affected sigmoid colon, unaffected proximal colon from UC patients, and sigmoid colon from healthy controls with an average of 1053 genes per cell were mapped into 21 clusters, six of which were epithelial cells (enterocytes, enterocyte progenitors, goblet cells, goblet progenitors, LGR5 + stem cells, and TRPM5 + tuft cells). A BEST4 + cell cluster was not uniquely identified but was found among transcripts in the enterocyte cluster.

## Colonic Microfold (M)-Like Cells

Microfold (M) cells are part of an integrated system of immunosurveillance in the intestinal mucosa, with a principal function of transporting luminal antigens to gut-associated lymphoid tissue (36, 37). This activity has led to the idea that M-like cells could contribute to a “leaky gut” (38). There are several types of small intestinal M cells that display a common set of morphologic and functional features but differ in their specific gene expression patterns. Our knowledge of M-cell morphologic, molecular and functional features are mainly based on small intestinal M cells. M-like cells in the colon have been described to emerge under inflammatory conditions in mouse models, but relatively little is known about M-like cells in human colon (39).

According to the Broad study, specific markers of colonic M-like cells in healthy tissue were quite different from M cells in ileal Peyer’s patches. Glycoprotein 2 (GP2), the M cell-specific marker that functions as a bacterial uptake receptor in the Peyer’s patches, was not reported in colonic M-like cells, and neither were genes such as PGLYRP2, CLEC7A (Dectin-1), nor JAG1. M cells from Peyer’s patches and colon shared only two transcripts, CCL20 and SPIB, which are known to initiate M cell differentiation (41). CCL20 is a UC GWAS gene that may serve as a marker of colonic M-cells in healthy tissue (40). Transcripts for both SPIB and TNFSF11, the RANKL receptor, in colonic M-like cells indicate similarities between the pathway of M cell differentiation in ileum and colon. However, SPIB was also present in BEST4 + and Tuft cells, precluding this gene as a specific M-like cell marker. SPIB transcription in BEST4 + cells was confirmed by the Oxford study (22).

We analyzed differences between M-like cells in healthy controls and UC (Table 2). Not only were M-like cell numbers significantly elevated in inflamed mucosa, these cells also had increased transcription of CCL20 and CCL23, implicating them in the recruitment of other immune cells and propagating inflammation. Only two transcripts, CCL20 and SPINK5, were elevated in M-like cells in healthy colon as well as in UC. However, both transcripts were highly upregulated in UC in other absorptive and secretory cell clusters, thus precluding them from being specific M-like cell markers. Notably, SOX8 did not appear upregulated in any other cell cluster in UC (Table 2). We conclude that SOX8 could be used to identify inducible colonic M-like cells and study their function in health and UC.

**TABLE 2:** Differential gene expression for M cells.

Broad Study	
Health	Ulcerative Colitis
**CCL23**	PI3
SOX8	FAM84A
**RP11-161M6.2**	CRABP2
NTRK2	KCNE3
**CCL20**	TMEM158
NPB	SLC6A14
MIA	ANKRD34A
**SPINK5**	HOXB13
SERPINF2	TCF12
KCNE2	BHLHE41
CTSE	
TNFAIP2	
**SPIB**	
MSLN	
TNFRSF11A	
POLD1	
SLC2A6	
AC026471.6	
TNFRSF11B	
AKR1C2	
GJB3	

	Transcript also reported in Oxford study (but not linked to M-cell)

**Red**	Transcript reported in at least one other cell lineage in Broad study

**Blue**	Transcript reported in both healthy subjects and UC in Broad study

## Goblet Cells

**FIGURE 2:**
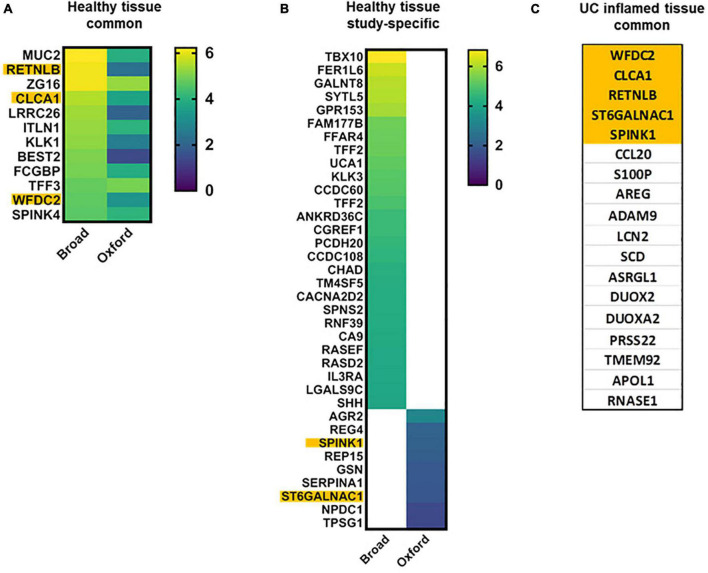
Goblet cell characteristic transcripts in health and UC inflammation. Heap maps of transcripts characteristic for differentiated and undifferentiated goblet cells in healthy tissue [**A,B**; log_2_(FC)]; transcripts characteristic for differentiated and undifferentiated goblet cells in UC **(C)** in both datasets. Transcripts in orange are common for healthy and UC-affected GCs. ITLN1 and CCL20 are GWAS genes.

Goblet cells are critical in maintaining a protective mucus layer that provides separation between epithelia and luminal content. Both studies identified undifferentiated and differentiated transcriptional clusters of goblet cells (GCs) in relationship to the colonic crypt-surface axis. GCs were sub-classified into immature or mature populations (Broad), or grouped into five transcriptionally distinct clusters (Oxford). Among the 64 major GC markers identified by the Oxford study, 35 were also found elevated in GC clusters in the Broad study. Twelve transcripts highly elevated (log_2_FC ≥ 4.0) in both, mature and immature GCs in Broad study were also elevated in this cell type in Oxford study (Figure 2). Both studies are concordant in that there is increasing expression of MUC2 and ZG16 along the crypt-surface differentiation axis, while TFF3, ITLN1, SPINK4, CLCA1, and WFDC2 expression is higher in immature GCs (Figure 2). Surprisingly, only BEST2 and ZG16 were GC-specific in Broad study.

Importantly, transcripts elevated in goblet cells (Figure 2) were in agreement with the subsequent study by Li et al. (35), including MUC2, ITLN1, REP15, LRRC26, NPDC1, TPSG1, SERPINA1, CLCA1, and SPINK4.

Of particular interest is the enrichment of mucins other than MUC2 in mature GCs. Both studies agree that transmembrane MUC1 and MUC4 are enriched in differentiated GCs, but are also detected in other cell clusters (transit amplifying (TA) cells). MUC12 is listed as elevated in GC cluster 4 in the Oxford study, but is elevated in colonocytes in the Broad study. MUC13 is enriched in mature GCs and in absorptive cells in both studies. MUC17 was not reported in GCs in any of the studies. Surprisingly, gel-forming MUC5B was elevated in mature GCs in the Oxford study but was not listed in GC clusters in the Broad study (instead, MUC5B was elevated in TA lineages).

The changes in goblet cells in UC through the prism of scRNA-seq are striking. It is widely accepted that number of GCs and the luminal mucus layer are significantly reduced in UC-damaged epithelia (41). Both studies found a significant decrease in GC numbers and significantly altered transcriptional signatures (Figure 2). However, the transcriptional sub-populations of spatially distinct goblet cells (crypt bottom vs. top) were preserved in active UC-derived epithelial tissues. Additionally, in the Oxford study, a novel cluster of inflammation-associated GCs was found in UC.

Overall, GC clusters in UC partially lose their secretory identity and are characterized by a mix of transcripts present in both absorptive and secretory lineages. Each GC cluster in UC exhibited highly heterogeneous transcriptional changes, and a consensus for GC markers was not evident. Among 139 transcripts listed in the Oxford study as upregulated in GCs in UC, only 15 (11%) were also found in the Broad study. Additionally, many of these transcripts were also significantly elevated in non-GCs in UC. This significant transcriptional transformation of GCs may explain their functional transformation.

In UC-affected epithelium (Figure 2), only SCD, RNASE1, and ASRGL1 were concordant with goblet cell findings by Li et al. (35).

STRING interactome analysis of GC-upregulated transcripts in UC from the Oxford study suggest that GCs gain properties for antigen presentation (HSA04612), stress response (GO:0006950), and immune regulation (GO:0002376). Other studies have alluded to this ability of GCs to act as antigen presenting cells, including delivery of antigens to dendritic cells (42) or microbial sensing and recruitment of inflammatory cells (43).

One of the most striking results from both scRNA-seq datasets is that MUC2 transcript levels were similar between healthy and UC samples, yet MUC2 protein is substantially decreased in patients with severe UC (41, 44). The discrepancy in detected MUC2 transcripts and protein in UC prompted us to perform a similar comparison for other goblet cell markers. Proteomic analysis of mucus from healthy or UC sigmoid colon biopsies showed that major core mucus components, including MUC2, FCGBP, CLCA1, and ZG16 were significantly reduced in active UC (42). Protein levels of TFF3, MUC4, 5B, 12 and 13 and ITLN1 did not change in mucus from UC patients. These data suggest post-transcriptional regulation of several core mucus proteins in disease accounts for differences between mRNA and protein expression in active UC.

It was surprising that transcript levels of MUC5AC, a gel-forming mucin that is typically associated with surface gastric epithelia, but can be induced in the intestine during infection (45, 46), was significantly elevated in inflamed colon. A recent study also found elevated MUC5AC transcript levels in active UC, although there was no significant correlation between MUC5AC expression levels and UC disease severity (47). However, MUC5AC was not reported among the core mucus proteins in active UC (45). MUC5AC is frequently present in colorectal adenomas and colon cancers, thus it’s elevation might be due to UC-induced carcinogenesis (48).

In conclusion, according to both scRNA-seq datasets, goblet cell clusters in UC lost some of their secretory hallmarks and were characterized by a mix of transcripts present in both absorptive and secretory lineages.

## Colonocytes

Colonocytes represent the largest cell pool in colonic epithelial tissue. Numerous studies have characterized the UC inflammation-induced pathologies of colonocytes and suggested that these pathologies must be pharmacologically addressed to achieve a full and sustained remission. Colonocytes can be spatially and functionally divided into three groups: Undifferentiated cells at the bottom of crypts, cells undergoing a transitional differentiation, and fully differentiated surface colonocytes. For the first time, scRNA-seq allows for precise characterization of the stages of cell differentiation and identification of the number of transcriptionally stable transition clusters which traverse from undifferentiated to fully differentiated. We analyzed the cluster specific markers of colonocytes in health and UC disease to gain insights into the molecular-cellular pathways driving UC disease.

### Differentiated Absorptive Colonocytes in Health and Ulcerative Colitis Disease

**FIGURE 3:**
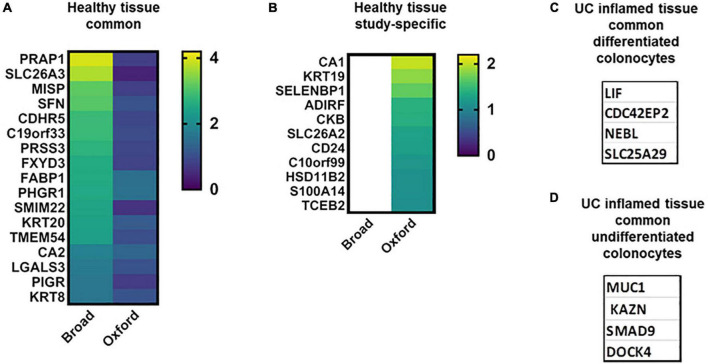
Colonocyte characteristic transcripts in health and UC inflammation. Heat maps of transcripts characteristic for differentiated and undifferentiated colonocytes in healthy tissue [**A,B**; log_2_(FC)]; colonocytes – characteristic transcripts in UC in both datasets **(C,D)**.

Both studies identified a cluster of terminally differentiated colonocytes (additionally to BEST4 + cells) named Crypt Top Colonocytes (CTC) in the Oxford study or Enterocytes in the Broad study. The CTC cluster included ∼ 264 upregulated transcripts (AUC > 0.7) and Enterocyte cluster included ∼150 significantly upregulated transcripts. Seventeen of these highly elevated transcripts are identified in both studies but neither of these transcripts is cluster-specific (Figure 3A). However, seven less elevated transcripts are cluster-specific and overlap in both studies (Supplementary Table 1A), and thus may serve as markers of mature colonocytes. ABCB1, an ATP-dependent drug efflux pump for xenobiotic compounds, is one of the common transcripts, and mutations in this gene are associated with IBD. Others are interferon-regulated proteins, including dsRNA-activated antiviral enzyme OAS1 which plays a critical role in cellular innate antiviral response, ubiquitin-like modifier ISG15, and sodium-phosphate symporter SLC20A1, which plays a fundamental housekeeping role in phosphate transport.

Many transcripts highly upregulated in differentiated colonocytes in one (Figure 3B) or both studies were also detected in other cell clusters (Supplementary Tables 1B,D,E). For example, CTC-specific CDHR2, CLCA4, MS4A12, and SLC9A3R1 were also significantly elevated in differentiated colonocytes in Broad study, but were not cluster-specific. SLC26A3/DRA, the bicarbonate/Cl- exchanger is one of these transcripts and is a well-known marker of differentiated colonocytes validated in numerous studies (49). Low levels of DRA are also detected in undifferentiated colonocytes in both studies. DRA is involved in response to stimulus, immune system process, cell junction organization, bicarbonate transport and regulation of sodium transport. The expression differences in such transcripts between the two studies is likely due to large differences in the number of analyzed cells (Table 1). A larger number of analyzed cells, as in Broad studies, improves the detection sensitivity for low abundance transcripts. Only a few transcripts are single cluster-specific and could not be detected in other clusters. Instead, the majority of the transcripts form gradients between different cell clusters. Further validation experiments are necessary to test whether inter-cluster copy number difference is large enough to allow use of a particular transcript as the cluster marker of differentiated colonocytes in healthy tissue, as in the case of DRA (Supplementary Tables 1B,D,E).

Only four transcripts were discordant (Supplementary Table 1). These transcripts were elevated in healthy Enterocytes in the Broad study but were among the UC inflammation-elevated transcripts in the Oxford study.

In UC inflammation, only four highly elevated and specific transcripts for differentiated colonocytes were common (Figure 3C and Supplementary Table 1I); these might serve as UC disease markers of the differentiated colonocyte cluster. In the Oxford study, the UC-affected CTC cluster is characterized by ∼300 upregulated transcripts, with only 17 transcripts shared between inflamed and healthy CTC (Supplementary Table 1D), suggesting substantial structural and functional changes in this cell type in disease. STRING analysis of the CTC-specific transcripts in UC confirmed high representation of immune system processes. In contrast, bicarbonate and sodium transport and cell junction organization pathways were absent from the UC transcriptome. Loss of SCL26A3 was previously linked to inflammatory diarrhea in UC patients (50, 51).

Additionally, there were significant changes in the composition of claudins in CTC cells; while CLDN7, 23, and 4 were elevated in healthy epithelium, CLDN1 and eight were elevated in disease. Mutations in NOD2, one of the major UC disease susceptibility genes, have been shown to alter the composition of tight junction proteins including upregulation of CLDN1 (52). scRNA-seq results confirm previous findings and correlate with the less studied CLDN8 in UC. UC-affected CTC cells also lose expression of CDHR5, an important intermicrovillar adhesion molecule that forms calcium-dependent heterophilic complexes with CDHR2 on adjacent microvilli (53). These two proteins control the packing of microvilli at the apical membrane of colonocytes and play a central role in brush border differentiation. Taken together, the CTC transcriptional signature reflects loss of differentiation in UC-inflamed epithelium. Interestingly, transcripts related to viral entry, specifically TMPRSS2, CHMP2A, 2B, and 4B, are downregulated in the CTC cluster in active UC, which might be protective against viral infections, including SARS-CoV2, despite an elevated cytokine profile (54, 55).

In the Broad study, ∼111 protein transcripts were upregulated in the UC-inflamed Enterocyte cluster with only 16 being cluster-specific (Supplementary Tables 1I,L). Again, large difference between the numbers of cluster specific elevated transcripts in UC–affected differentiated colonocytes between the studies is probably due to the number of analyzed cells; the less cells are analyzed the more cluster-specific transcripts it would produce. Elevation of the proinflammatory cytokine LIF was previously reported in UC patients, confirming the scRNA-seq data. LIF stimulates cell proliferation and thus may play a critical role in tumor development in UC patients (56). Another 13 transcripts elevated in multiple clusters in UC from the Broad study were also specifically elevated in UC-affected CTC cells (Supplementary Table 1J). The Broad study identified a single SAA1 transcript elevated in both healthy (specifically) and UC-affected (non-specifically) Enterocytes. SAA1 expression in epithelium has been shown to serve as an important link between mucosal T cells, microbial communities, and their tissue environment in patients with IBD (57), further supporting these scRNA-seq findings.

### Undifferentiated Colonocyte Clusters in Health and Ulcerative Colitis Disease

The pool of immature healthy colonocytes is mainly represented by three distinct clusters of Immature Enterocytes 1 and 2, and Enterocyte Progenitors in the Broad study. We searched for specific cluster markers among the substantially elevated protein coding transcripts (log2fc ≥ 4.0; Supplementary Table 2). DUOX2 and TMEM150B were exclusively elevated in the healthy Immature Enterocytes 1 cluster. Similarly, FOXH1, C14orf180, and HS3ST6 may serve as markers of Immature Enterocytes 2. Surprisingly, no protein transcripts elevated above this threshold were among Enterocyte Progenitors. In addition, there were no transcripts upregulated above the threshold that are common and specific for all three clusters.

In UC-inflamed tissue, according to the Broad study, eight elevated (log2fc ≥ 4.0) transcripts were shared among all three clusters (Supplementary Table 2H). However, these transcripts were upregulated in numerous other clusters, including differentiated absorptive and secretory lineages. Importantly, the expression pattern of these eight transcripts substantially differs between inflamed UC and healthy tissue. In healthy tissue, they are upregulated in a single or few clusters. However, in UC they are spread across many clusters, further demonstrating that the major UC disease phenotype dissociates the molecular boundaries between different cell types and their stage of differentiation. For example, CCL20 appears as an M-like cell marker in healthy tissue, but is broadly elevated in both secretory and absorptive cells in UC. Similarly, in healthy epithelium, GLDN and DUOX2 are elevated exclusively in Enterocyte Progenitors and Immature Enterocytes 1, respectively, but in UC are elevated in at least seven other secretory and absorptive cell clusters. DUOX2 is the only Immature Enterocytes 1 transcript elevated in both healthy and diseased epithelium. It has been shown that epithelial DUOX2 and DUOXA2 form the predominant enzyme system for H_2_O_2_ production in human colon and in active UC (58), further supporting these scRNA-seq data.

Elevated MUC1 could be considered as a marker of Immature Enterocytes 1 in active UC (Figure 3D), while in healthy tissue MUC1 is expressed throughout all epithelial clusters. SMAD9, KAZN, and DOCK4 (Figure 3D) could be considered as markers of Immature Enterocytes 2. Several transcripts highly elevated in the clusters of undifferentiated colonocytes further illustrate the loss of cluster-specificity expression in inflamed UC epithelium compared to healthy tissue. For example, in healthy epithelium, SDR16C5 is elevated in M-like cells, Goblet cells and Tuft cell clusters, MIA is elevated in M-like and Tuft cells; and PRSS22 is elevated in Secretory and Tuft cells; however, all three transcripts are elevated in nearly all the epithelial cell clusters in UC. Importantly, SI, a well-known marker of fully differentiated small intestinal enterocytes was not detected in healthy human colon, but was broadly expressed in UC, including undifferentiated colonocytes. SI has been detected in inflammatory, regenerative, and dysplastic mucosa in Ulcerative Colitis (59). Additionally, OLFM4 is mainly upregulated in Stem and TA1 clusters in healthy epithelium, but in UC is elevated in TA and Absorptive Enterocytes. The expression pattern of these transcripts again shows the loss of cell type specificity in UC.

The transcriptional comparison of immature colonocytes between two studies is challenging and not straightforward due to differences in transcript clustering algorithms. We speculated that Colonocytes and Absorptive Progenitors could be clusters that represent immature colonocytes in Oxford study. Eight transcripts found to be most elevated in all three undifferentiated colonocyte clusters in the Broad study were also significantly elevated in UC Colonocytes in Oxford study. Four transcripts (TNIP3, S100P, DUOX2, and CXCL1) were also elevated in Absorptive Progenitors. Again, similar to the results of the Broad study, none of the transcripts were cluster-specific and all were upregulated in most clusters except EECs in the Oxford study. Additionally, both datasets suggest that DOCK4, a membrane-associated cytoplasmic protein, which functions as a guanine nucleotide exchange factor involved in regulation of adherence junctions between the cells, may be a reliable marker of undifferentiated colonocytes in UC disease. Although its role in UC disease is not known, DOCK4 was recently identified as a regulator of goblet cell differentiation and MUC2 production in the gut (60).

Several transcripts identified as markers of healthy differentiated colonocytes in the Broad and Oxford studies (VAMP8, OAS1, ISG15, SLC20A1; Figure 3) were concordant with data by Li et al. However, the remaining transcripts that were listed as markers of healthy differentiated or undifferentiated colonocytes (Supplementary Tables 1, 2) were not reported. CDC42EP2, a potential marker of UC-affected differentiated colonocytes was also reported by Li et al., while MUC1, a potential marker of undifferentiated colonocytes, was elevated in tuft cells in addition to enterocyte progenitors.

## Colonic Stem Cells

Altered histology and transcriptional profiles in UC epithelia have been observed to persist even after achieving a disease remission (61), suggesting that the colonic stem cell (SC) may be permanently changed during the course of disease (18). Somatic nonsense or gene mutations have been discovered in UC epithelia, including genes involved in IL-17 signaling, such as NFKBIZ, ZC3H12A, and PIGR (62). Inflammation-induced somatic mutations might provide clonal advantage to these stem cells and propagate UC-related pathologies. We next analyzed stem cell profiles in both scRNA-seq data sets to search for potential signatures associated with UC.

The SC cluster from healthy tissue was not described in the Oxford study, possibly because only 38 cells contained the stem cell marker LGR5. The Broad study identified LGR5 as the most SC-enriched and specific transcript (Table 3). Among the 18 most elevated SC transcripts (log_2_FC ≥ 4.0), 16 were SC specific, which might substantially broaden the ability to identify and characterize human colonic SC *via* immunoblotting, immunofluorescence, functional assays and other approaches. OLFM4 is often used as a substitute marker for SC (63) and is elevated (log_2_FC 2.4) in both SC and TA1 cells. Interestingly, several other highly expressed SC-specific transcripts are lincRNAs (RP11-219E7.4 and RP11-760H22.2) and RP11-84C10.4 antisense, all of which might be involved in SC-specific regulation of CFTR.

**TABLE 3:** Differential gene expression for stem cells.

Health		Ulcerative colitis	
Highly expressed transcripts reported in Oxford Study	Highly expressed transcripts reported in Broad Study	Highly expressed transcripts reported in Oxford Study only	Highly expressed transcripts reported in Broad Study only
None reported	LGR5	EZR	**FN1**
	RP11-219E7.4	NFKBIA	**SI**
	CYP4 × 1	C15orf48	**SPON1**
	OXGR1	JUNB	**B3GNT7**
	SMOC2	IER2	**KCTD12**
	NR0B2	IFI27	**HOXB13**
	SLC39A2	HBEGF	**MT-TV**
	SHISA9	FAM3D	**SLC28A2**
	PTPRO		**PRAC1**
	RP11-84C10.4		
	CAPN6		
	RGMB		
	FMN2		
	CELF5		
	**TERT**		
	RP11-760H22.2		
	**ASCL2**		
	C1orf95		

	lnc RNA		

	Transcript is downregulated in stem cells in UC		

	Transcript is elevated in other healthy cell clusters		

	Transcript is elevated in healthy stem cells		

**Red**	Transcript is elevated in other cell types in UC		

**Blue**	Transcript identified in Genome-Wide Association Study (GWAS)		

In the Broad study, 65 transcripts were upregulated in SC in active UC relative to healthy controls. Although a majority of these transcripts were also upregulated at least in one other cell cluster, eight transcripts were specific to SC in UC (Table 3). Surprisingly, the upregulated transcripts are commonly associated with healthy differentiated cell types such as colonocytes (EZR, IFI27) or goblet cells (IER2, HBEGF, NFKBIA, and FAM3D). The functional significance of these UC-induced SC transformations remains to be determined.

STRING analysis of the 65 upregulated SC transcripts found associations to stress response (GO:0006950) and cell communication (GO:0010646) as the most inclusive gene ontology pathways. These pathways also included a large set of molecules involved in immune response. Multiple HLA and IFI transcripts, either SC-specific or shared between SCs and other epithelial cell clusters in UC, were significantly elevated, indicating a shift in SC function toward antigen presentation in UC. HSPA6 and CCL20 are UC GWAS genes, possibly linking genetic predisposition to UC with changes in the SC transcriptome. In UC, the fate of SC markers LGR5, OLFM4, or AXIN2 is not clear, as these transcripts were not listed among those enriched or downregulated in UC in the Oxford study. According to the Broad study, AXIN2 was significantly downregulated in stem cells in UC. In contrast, OLFM4 was slightly upregulated in stem cells and much more elevated in all TA clusters and in Enterocytes in UC. The lack of overlap between the two studies on SC markers in inflamed colonic tissue indicates that it is too soon to hypothesize on the mechanism(s) contributing to long-lasting changes in SCs in active UC.

The expression of potential stem cell-enriched transcripts (Table 3) was not reported in healthy epithelium by Li et al., despite the presence of LGR5 + stem cell clusters in both healthy and UC tissues. In UC, markers elevated in stem cell cluster in either the Oxford or Broad study were discordant with the findings reported by Li et al. (35). EZR, a classical marker of epithelial cells, was found elevated in several epithelial cell clusters, and surprisingly, in fibroblasts. IFI27 was non-specifically elevated in stem cells, while FAM3D and PRAC1 were elevated in stem cells when expression was normalized to the proximal colon transcriptome but not to healthy sigmoid colon. NFKBIA, JUNB, and IER2 were elevated in other epithelial and/or non-epithelial cell clusters, but not in LGR5 + stem cells.

## Discussion

RNA-seq of single cells paves the way for discovery of novel epithelial cell clusters in UC. The major differences between the two studies were in the methods and protocols used to isolate cells and the large difference in the number of analyzed cells. In addition, patient selection is also variable and patients are not comparable across studies (Table 1). These limitations should not deter researchers from using the findings in these studies, but rather emphasize the need to validate markers by alternative techniques. The Broad and Oxford studies make important contributions to defining the UC epithelia transcriptional landscape, but points of incongruity suggest that expanded profiles from additional subjects are necessary to refine disease-associated hypotheses. Combining scRNA-seq with other -omics into a reference disease atlas would provide a powerful tool to iteratively model, test, and revise mechanistic models in UC experimental settings for drug testing and other clinical studies.

Because parameters vary widely across studies, it is not straightforward to determine whether a particular transcript is specifically changed within a single cluster and thus be a reasonable target for further evaluation in human tissue or organoid models. Our analysis acknowledges this gap and identifies some areas in which non-omics validation is important.

scRNA-seq in the Broad and Oxford studies revealed that transcriptional signatures and cell type boundaries become more nebulous in UC. Multiple transcripts were differentially regulated across many clusters, challenging the classical concept of unique cell cluster markers associated with active UC. The lack of clearly separated transcripts reflects a general loss of cell specialization, replaced by cell phenotypes that do not map to healthy tissue. Evaluating markedly altered transcripts in relation to known interactors (interactome analysis) adds depth to cluster analysis, but these strategies are not useful if cell type clusters lack discrete boundaries in disease.

Study interpretations are limited by the heterogeneity of subjects (including gender, age, ethnicity, site of colon biopsy, stage of UC disease, and prior use of anti-inflammatory therapies). The total number of cells acquired and transcripts sequenced also contribute to variability in sensitivity for detecting rare cell types or transcripts. Validation of findings at the protein level is also imperative to advance understanding of the molecular features conserved in UC epithelia.

## Author Contributions

JS, JF-A, and OK researched data, contributed to discussion of content and writing, generated figures and tables, and reviewed/edited the manuscript before submission. WH and JH contributed to discussion of content for the article and reviewed/edited the manuscript before submission. JI researched data for the article and reviewed/edited the manuscript before submission. All authors contributed to the article and approved the submitted version.

## Conflict of Interest

The authors declare that the research was conducted in the absence of any commercial or financial relationships that could be construed as a potential conflict of interest.

## Publisher’s Note

All claims expressed in this article are solely those of the authors and do not necessarily represent those of their affiliated organizations, or those of the publisher, the editors and the reviewers. Any product that may be evaluated in this article, or claim that may be made by its manufacturer, is not guaranteed or endorsed by the publisher.

## Footnotes

1
https://string-db.org/
